# Prevention of Acute Upper Respiratory Infections by Consumption of Catechins in Healthcare Workers: A Randomized, Placebo-Controlled Trial

**DOI:** 10.3390/nu12010004

**Published:** 2019-12-18

**Authors:** Daisuke Furushima, Takuma Nishimura, Norikata Takuma, Ryo Iketani, Tomohito Mizuno, Yuji Matsui, Tohru Yamaguchi, Yu Nakashima, Shinji Yamamoto, Masanobu Hibi, Hiroshi Yamada

**Affiliations:** 1 Department of Drug Evaluation and Informatics, Graduate School of Pharmaceutical Sciences, University of Shizuoka, Shizuoka 422-8526, Japan; m14086@u-shizuoka-ken.ac.jp (T.N.); s16805@u-shizuoka-ken.ac.jp (R.I.); hyamada@u-shizuoka-ken.ac.jp (H.Y.); 2 White Cross Nursing Home, Tokyo 189-0021, Japan; hide1844@wa2.so-net.ne.jp; 3 Biological Science Research Laboratories, Kao Corporation, Tokyo 131-8501, Japan; mizuno.tomohito@kao.com (T.M.); matsui.yuji@kao.com (Y.M.); hibi.masanobu@kao.com (M.H.); 4 Health Care Food Research Laboratories, Kao Corporation, Tokyo 131-8501, Japan; yamaguchi.tohru@kao.com; 5 Personal Healthcare Research Laboratories, Kao Corporation, Tokyo 131-8501, Japan; nakashima.yuu@kao.com (Y.N.); yamamoto.shinji@kao.com (S.Y.)

**Keywords:** acute upper respiratory infection, catechins, epigallocatechin gallate, influenza like illness, randomized controlled trial

## Abstract

Catechins, phytochemicals contained mainly in green tea, exhibit antiviral activity against various acute infectious diseases experimentally. Clinical evidence supporting these effects, however, is not conclusive. We performed a placebo-controlled, single-blind, randomized control trial to evaluate the clinical effectiveness of consumption of catechins-containing beverage for preventing acute upper respiratory tract infections (URTIs). Two hundred and seventy healthcare workers were randomly allocated to high-catechin (three daily doses of 57 mg catechins and 100 mg xanthan gum), low-catechin (one daily dose of 57 mg catechins and 100 mg xanthan gum), or placebo (0 mg catechins and 100 mg xanthan gum) group. Subjects consumed a beverage with or without catechins for 12 weeks from December 2017 through February 2018. The primary endpoint was incidence of URTIs compared among groups using a time-to-event analysis. A total of 255 subjects were analyzed (placebo group *n* = 86, low-catechin group *n* = 85, high catechin group *n* = 84). The URTI incidence rate was 26.7% in the placebo group, 28.2% in the low-catechin group, and 13.1% in the high-catechin group (log rank test, *p* = 0.042). The hazard ratio (95% confidence interval (CI)) with reference to the placebo group was 1.09 (0.61–1.92) in the low-catechin group and 0.46 (0.23–0.95) in the high-catechin group. These findings suggest that catechins combined with xanthan gum protect against URTIs.

## 1. Introduction

Among the general population, acute upper respiratory tract infections (URTIs) are the most common infectious diseases, generally occurring two to five times a year, and are a leading cause of work or school absences [1,2,3]. URTIs are caused by various viruses such as respiratory syncytial virus, adenovirus, rhinovirus, and influenza virus interacting with the mucosa of the upper airways [4,5]. Although URTIs are usually associated with mild symptoms, the primary treatment is aimed at relieving pain or decreasing a high temperature [6,7]. UTRI viruses spread easily via contaminated droplets and contact, and there are no effective preventive measures. Healthcare workers, in particular, are constantly exposed to viral respiratory infections in medical facilities [8]. Although frequent hand washing, wearing face masks, and avoiding crowds are recommended for prevention, there is no clinical evidence supporting a preventive effect of these measures [9]. Therefore, developing new approaches with nonpharmaceutical preventive interventions available to the general public or facility workplaces is an important challenge in public health.

Catechins are a mixture of compounds classified as flavanols found in green tea, *Camellia sinensis*, that include the following: epicatechin (EC), epicatechin gallate (ECg), epigallocatechin (EGC), and epigallocatechin gallate (EGCg), as well as their thermal isomers, that is, catechin (C), catechin gallate (Cg), gallocatechin (GC), and gallocatechin gallate (GCg) [10,11,12]. EGCg, a major green tea catechin, exhibits broad-spectrum antiviral, antibacterial, and inhibitory effects against various viral families, including *Retroviridae*, *Flaviviridae*, *Herpesviridae*, *Hepadnaviridae*, *Adenoviridae*, and *Orthomyxoviridae,* in vitro [13]. Catechins, especially EGCg, also inhibit replication of the influenza virus in cell culture and may have direct antiviral effects [14,15,16,17]. Although the mechanisms of these effects of EGCg are not fully understood, EGCg appears most likely to interfere with viral membrane proteins, thereby inhibiting the early stage of infection, for example, attachment, entry, and membrane fusion [18]. Several clinical studies of green tea consumption, administration of catechins from green tea extract, or gargling green tea for infectious disease, such as seasonal influenza infection or common cold, prevention have been reported. In a placebo-controlled, randomized study, Rowe et al. [19] demonstrated that adults consuming capsules containing the mixture of EGCg and L-theanine twice daily for 3 months had 32.1% fewer cases of common cold or influenza symptoms and 35.6% fewer symptom days compared with those taking a placebo capsule. Park et al. [20] reported that the number of cups of green tea consumed per day or per week was inversely associated with the incidence of seasonal influenza in school-age children. Conflicting results have been reported from the placebo-controlled, randomized studies that confirmed the gargling with green tea catechins on prevention of seasonal influenza infection in healthy adults or healthcare workers [21,22]. Although several experimental studies have demonstrated the antiviral activity of catechins, there are relatively few clinical studies, and most studies of green tea consumption (capsules) or green tea gargling have been performed in preliminary studies which have insufficient power calculation, including a small number of participants, and do not provide conclusive evidence [19,22,23,24,25]. In particular, most clinical studies evaluating the effects of consuming capsules containing green tea catechins or gargling green tea toward the prevention of various infectious diseases focused on seasonal influenza infection, and relatively few studies evaluated URTIs. Therefore, the clinical effects of catechins for the prevention of URTIs have remained largely unexplored.

In the present study, we evaluated the clinical efficacy of consuming catechins-containing beverage for preventing URTIs in healthy adults working at a healthcare institute.

## 2. Materials and Methods

### 2.1. Subjects and Study Design

This study was a randomized, placebo-controlled, single-blind trial performed over 12 weeks during the winter season, from 1 December 2017 through 28 February 2018 in Higashimurayama city, Tokyo, Japan. Recruitment was started in 16 September 2017 using emails and posters describing the outline of the study. The subjects were recruited from among healthcare workers at the White Cross Nursing Home, Tokyo White Cross Hospital, and Tokyo Bannan Hakuko-En Elderly Care Facility (Higashimurayama, Tokyo, Japan).

The exclusion criteria were the following: systemic chronic infectious disease hepatic dysfunction, kidney disease, any other serious disease; immune deficiency or autoimmune disease; taking drugs influencing immune function; allergy to tea-derived components; those considered unsuitable for participation in the study by the study physician. Compatibility of these criteria was assessed by an investigator using a questionnaire. Study subjects were randomly assigned to one of three subject groups: high-catechin group (consuming catechins-containing beverages three times daily), low-catechin group (consuming a catechins-containing beverage once daily), or a placebo group (consuming a placebo beverage once daily), at a ratio of 1:1:1 after screening and baseline evaluations, according to a predetermined randomization code that was computer-generated at the Osaka University Graduate School of Medicine (Osaka, Japan). The randomization was conducted using a permuted block method with a block size of six and stratified according to the participants’ facility. Intervention samples, catechins beverage or placebo beverages, were supplied individually under blinded conditions (to subject), and the allocation to each group was concealed by a randomized number code. Group allocation was concealed from the investigator who carried out the monitoring during the intervention.

### 2.2. Ethical Considerations

The present study was performed according to the principles of the Declaration of Helsinki and Ethical Guidelines for Medical and Health Research Involving Human Subjects. The study protocol was approved by the ethics committee of the University of Shizuoka (No. 29-20, 15 September 2017) and Kao Corporation (10 August 2017). All subjects provided written informed consent prior to participating in the study. This trial was registered with the University Hospital Medical Information Network (UMIN; http://www.umin.ac.jp/; Registration No. UMIN000030103) on 30 November 2017.

### 2.3. Intervention

Subjects were provided catechins-containing beverages or placebo beverages over the course of 12 weeks. During the intervention period, the subjects were instructed to drink the beverage one or three times per day. Table 1 shows the catechin composition of each beverage, which was analyzed using the HPLC method described by Saijo and Takeda [26]. The catechins-containing beverage contained a total of 57 mg of catechins (including 20 mg EGCg) and 100 mg of xanthan gum, to increase the viscosity, acidifiers, flavoring agents, vitamins, and sweeteners or a placebo beverage containing 0 mg catechins and 100 mg xanthan gum, acidifiers, flavoring agents, vitamins, and sweeteners was manufactured by the Kao Corporation (Tokyo, Japan). The catechins and placebo beverages were manufactured to be indistinguishable by taste, smell, and appearance. Each group received a powder, which had to be dissolved by the subjects in 40 mL water. These beverages had to be consumed immediately. The subjects were prohibited from discussing or otherwise communicating about the test beverages or intervention with each other. From the time of enrollment until the end of the study, the subjects were instructed to orally ingest one cup of the beverage one or three times each day. In the low-catechin group, the beverage was consumed in the morning before going to work, and in the high-catechin group, the beverage was consumed in the morning before going to work, after lunch, and in the evening before going home from work. The subjects were instructed to avoid foods or drugs that influence immune function and to avoid changing their lifestyle, including diet or exercise, as much as possible during the intervention period.

### 2.4. Outcome Assessment and Clinical Monitoring

For all eligible subjects, age, sex, body mass index (BMI), seasonal influenza vaccination, hand-washing habits, gargling habits, and green tea drinking habits (drinking more than 100 mL green tea/day) were collected using a questionnaire as the baseline clinical characteristics. The primary endpoint was incidence of URTIs during the 12-week intervention period. A URTI diagnosis was defined as a doctor-diagnosed URTI on the basis of one or more respiratory symptoms (cough, sore throat, and/or shortness of breath) as well as one or more systemic symptoms (self-reported fever (temperature ≥37.8 °C, headache, myalgia, and/or malaise). The secondary endpoints were as follows: (1) incidence of influenza-like illness (ILI) during the intervention period, (2) degree of physical symptoms of URTIs, and (3) frequency and severity of adverse events during the intervention period. An ILI was defined as the presence of fever (temperature, ≥37.8 °C) and any two of the following clinical symptoms: cough, sore throat, headache, and myalgia through subject-reported measures [27], or positive results of a rapid antigen detection test for seasonal influenza at a medical institution [28]. The degree of physical symptoms of URTIs was assessed as nasopharyngeal symptoms (sneezing, runny nose, stuffy nose), hypopharyngeal symptoms (scratchy throat, dry throat, sore throat), and systemic symptoms (fatigue, chills, joint pain due to cold symptoms, headache). These symptoms were self-assessed by each subject according to a five-step rating scale questionnaire (0, nothing; 1, a little; 2, somewhat; 3, quite; and 4, terrible). For collecting the data of the primary and secondary endpoints, each subject was required to fill out a daily questionnaire concerning the occurrence of URTIs and intervention compliance during the intervention period. For evaluation of the safety of the intervention, adverse events were monitored throughout the intervention period. At the end of the intervention period, all subject data were evaluated by two independent physicians to determine the presence or absence of adverse events. Furthermore, regular monitoring was conducted every 4 weeks, and the responses to the questionnaires by each subject were carefully checked for missing data, intervention compliance, and occurrence of adverse events throughout the study.

### 2.5. Sample Size Calculations

Sample size calculations assumed an overall two-tailed alpha of 0.05 to be equally distributed between the comparisons of high-catechin and low-catechin groups vs. placebo. Sample size calculations were performed on the basis of previous reports of catechins [25], assuming a 20% infection rate for URTIs during the winter season, and the efficacy of catechin consumption was estimated to be 67.5%. A sample size of 95 subjects in each group (total 285 subjects) was estimated to have at least 80% power to detect the difference in the prevention effect as the primary endpoint with a significance level of 0.05.

### 2.6. Statistical Analysis

For the primary endpoint analysis, the number of URTI events was summarized, and a chi-squared test of independence was applied to compare the frequencies of URTI events among groups. The time-to-event method, Kaplan–Meier survival curves, log-rank test, and Cox proportional hazards model were used to compare the three groups with respect to the cumulative UTRI incidence rate, and the hazard ratio and corresponding 95% confidence intervals (CIs) were estimated. For multivariate analysis, the Cox proportional hazards model adjusted for age, sex, smoking habit, and compliance rate was adopted. For secondary endpoint analysis, differences in the degrees of symptoms associated with URTIs in each group were compared by analysis of variance (ANOVA) and Dunnett’s method. The occurrence of adverse events was summarized and presented with descriptive statistics. Descriptive statistics for all analyses are expressed as means, standard deviations (SD), and standard error of the mean (SEM) for continuous variables, and as absolute and relative frequencies for nominal and ordinal variables. All analyses were conducted based on the intention-to-treat principle, and statistical analysis was conducted using the statistical analysis program R (version 3.4.2, R Development Core Team 2018, R Foundation for Statistical Computing, Vienna, Austria). A *p* value of less than 0.05 was considered to indicate statistical significance.

## 3. Results

### 3.1. Subjects

**Figure 1:**
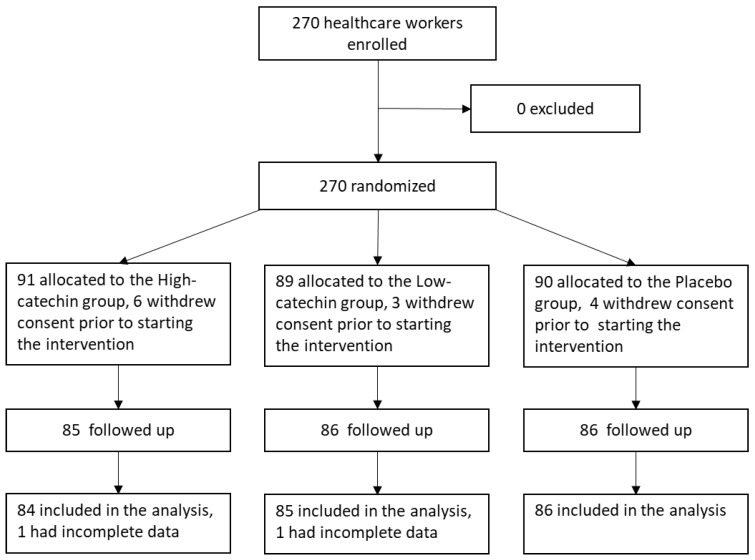
Flow diagram of the trial.

A flow diagram of the trial is shown in Figure 1. Although it was slightly less than the estimated number of subjects, a total of 270 subjects were assessed for eligibility and enrolled into the study. Of those, 91 subjects were assigned to the high-catechin group, 89 subjects were assigned to the low-catechin group, and 90 subjects were assigned to the placebo group. In the high-catechin group, 84 of 91 subjects were included in the analysis set. In the low-catechin group, 85 of 89 subjects were included in the analysis set. In the placebo group, 86 of 90 subjects were included in the analysis set. Thus, the data from 255 subjects were available for the analysis. The baseline characteristics of the subjects according to group are summarized in Table 2. Mean ± SD subject age was 43.2 ± 12.1 years, and 196 of 255 subjects (76.9%) were female. The rate of seasonal influenza vaccination was 95.7% (244 of 255 subjects), hand washing habit was 97.6% (249 of 255 subjects), and hand disinfection habit was 62.7% (160 of 255 subjects). Baseline values did not differ significantly among the three groups. Mean ± SEM intervention compliance rates were 87.0 ± 1.1% in the high-catechin group, 91.9 ± 1.0% in the low-catechin group, and 92.5 ± 0.7% in the placebo group.

### 3.2. Incidence of Acute Upper Respiratory Tract Infections and Influenza-Like Illness

**Figure 2:**
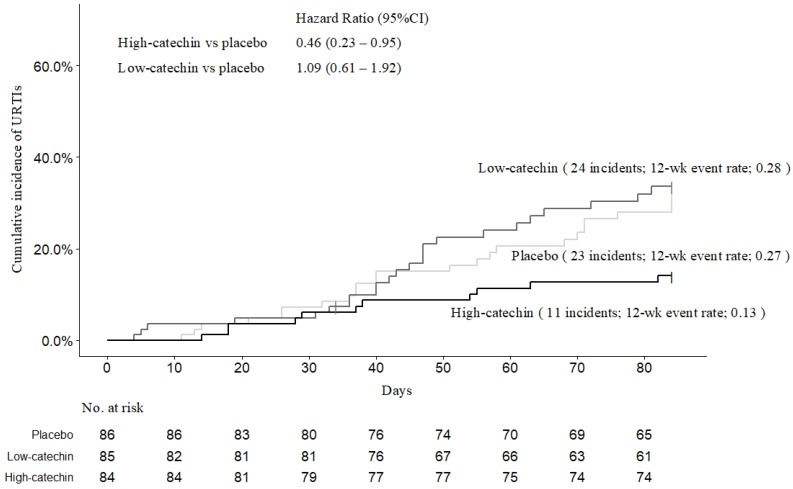
Kaplan–Meier estimates of URTI incidence for each group. CI denotes confidence interval.

The URTI incidence (primary endpoint) during the 12-week intervention period was 11 cases in the high-catechin group, 24 cases in the low-catechin group, and 23 cases in the placebo group. Figure 2 shows the Kaplan–Meier results for the cumulative URTI incidence in the three groups. The estimated 12^th^ week cumulative incidence was 13.1% for the high-catechin group, 28.2% for the low-catechin group, and 26.7% for the placebo group (log-rank test: overall, *p* = 0.04, low-catechin vs. placebo, *p* = 0.77, high-catechin vs. placebo, *p* = 0.03) The cumulative URTI incidence at the fourth and eighth weeks was 6.0% and 10.7% in the high-catechin group, 4.7% and 21.4% in the low-catechin group, and 7.0% and 16.3% in the placebo group, respectively. Compared with the placebo group, the cumulative URTI incidence was significantly lower in the high-catechin group (hazard ratio, 0.45; 95% CI, 0.22 to 0.92), but not in the low-catechin group (hazard ratio, 1.09; 95% CI, 0.61 to 1.92). Multivariate Cox hazard regression model analysis adjusted for age, sex, smoking habit, and compliance rate of test beverage ingestion showed a significantly lower URTI incidence in the high-catechin group (adjusted hazard ratio, 0.37; 95% CI, 0.18 to 0.78). The risk ratio (95% CI) of the low-dose group to the placebo group was 1.06 (0.65 to 1.72), and that of the high-dose group was 0.49 (0.26 to 0.94; chi-squared test: overall, *p* = 0.04). The incidence of ILI (secondary endpoint) was a total of 10 cases (3.9%) overall, 1 case (1.2%) in the high-catechin group, 5 cases (5.9%) in the low-catechin group, and 4 cases (4.7%) in the placebo group. The incidence rate seems lower in the high-catechin group, but statistical analysis was not performed due to the small number of cases.

The symptom degree score for nasopharyngeal, hypopharyngeal, and systemic symptoms for each group is shown in Table 3. The mean nasopharyngeal symptom score was significantly lower in the low-catechin group and the high-catechin group compared with the placebo group (high-catechin group vs. placebo; *p* = 0.02, low-catechin group vs. placebo; *p* = 0.02, Dunnett’s test). The mean hypopharyngeal symptom score tended to be lower in the low-catechin group and the high-catechin group than in the placebo group, but the differences were not statistically significant (high-catechin group vs. placebo; *p* = 0.18, low-catechin group vs. placebo; *p* = 0.08; Dunnett’s test). The mean systemic symptom score did not differ significantly among the three groups (high-catechin group vs. placebo; *p* = 0.89, low-catechin group vs. placebo; *p* = 0.79; Dunnett’s test).

### 3.3. Adverse Events

Adverse events excluding the outcome-related events were determined by reviewing diaries recorded by the subjects during the trial period (Table 4). In total, subjects in the high-catechin group reported 24 adverse events, subjects in the low-catechin group reported 14 adverse events, and subjects in the placebo group reported 17 adverse events during the 12-week intervention period. The principal investigator judged these symptoms to be mild and temporary, and not related to ingestion of the test beverage on the basis of the symptoms, symptom duration, symptom degree, and outcome. The total number of adverse events was not significantly different among the three groups (chi-squared test, *p* = 0.28).

## 4. Discussion

The findings of the present study indicated that the URTI incidence was significantly lower in the high-catechin group than in the placebo group. After the 12-week intervention, the hazard ratio for the high-catechin group was 0.46 (95% CI, 0.23 to 0.95), indicating that the risk of URTI was reduced to about half by ingesting a catechins-containing beverage three times daily. These protective effects of consuming catechins against UTRIs were stronger than those of consuming catechins against influenza infection reported in an epidemiological study [20] and by taking catechins capsules in an intervention trial [25]. The reason for the difference may be the increased viscosity by the addition of xanthan gum, a type of polysaccharide, which may prolong the retention time of the catechins in the nasopharyngeal area, but the details are unknown and further research is needed. Moreover, in this study, ingestion of catechins by healthcare workers had preventive effects on the development of acute URTIs. Healthcare workers may have a higher risk of URTIs due to their close contact with sick people, and the prevalence of UTRIs in healthcare workers may be much higher than that in the general population. Therefore, the effects of catechins might be even more pronounced in the general population than in the subjects in this study.

The degree of the nasopharyngeal symptoms during URTIs, as assessed by the subjects, was also significantly lower in the high-catechin group than in the placebo group. The difference between the low-catechin group and the placebo group was not statistically significant. We were unable to evaluate the effectiveness of catechins against ILI because the incidence of ILI was too low for statistical analysis. The incidence rate of ILI was 3.7% overall, which was approximately half that of the general population in Japan during the 2017–2018 season, as estimated from the nationwide seasonal influenza surveillance by the National Institute of Infectious Diseases, Japan [29]. Thus, the protective effects of catechins against ILI remain unclear, and further studies are required.

The present study demonstrated a significant difference in URTIs between the high-catechin group, but not the low-catechin group, and the placebo group. The amount of catechins ingested in the high-catechin group was equivalent to that in approximately one cup of green tea, when converted to the average catechin intake from consuming regular green tea, but the amount of catechins ingested in the low-catechin group was less than one cup of green tea per day [30]. The clinical antiviral effects are reported to be dose-dependent. Park et al. reported that the number of cups of green tea consumed per day is inversely associated with the incidence of seasonal influenza (odds ratio with the consumption of 1 to 3 cups/day or 3 to 5 cups/day compared with 1 cup/day were 0.62 (95% CI, 0.41 to 0.95) and 0.54 (95% CI, 0.30 to 0.94), respectively) [19]. One potential reason for this discrepancy is the duration of the presence of the catechins in the oral cavity and on the mucous membranes. In general, catechin solutions are held for only a very short time in the oral cavity and mucosa, and the duration may not be sufficient for effective virucidal activity against microorganisms and viruses. Tamura et al. [31] reported that adding a mixture of polysaccharides to catechins extended their antibacterial effects in the oral cavity. In the present study, the virucidal activity was enhanced by adding xanthan gum, a kind of polysaccharide, to the catechins-containing beverage in an effort to extend the duration of the presence of the catechins in the oral cavity or mucous membrane. Long-term maintenance of virucidal activity could be related to the duration of presence of catechins in the oral cavity or upper airway mucosa and the chemical stability. The duration of catechin effects in vivo may also contribute to the differences observed between the high-catechin and low-catechin groups. The high-catechin group ingested the catechins-containing beverage three times a day, while the low-catechin group ingested the catechins-containing beverage only once a day before going to work in the morning. These findings might indicate that the timing of the catechin intake is also important for the virucidal activity in humans. Many virus infections, including influenza and the common cold, more commonly cause infection in the winter, in low-temperature seasons [32,33]. The circadian timings of infections, however, are unknown, and our understanding of the timing of infection during the day is limited [34,35]. Our study may provide a new insight into the circadian timing of viral infections in humans.

This study has some limitations. First, although the intake of foods or drugs that influence immune function was restricted during the study period, the effects of diet or exercise in daily life was not considered. Moreover, the baseline amounts of macronutrients and dietary polyphenols ingested by the participants were not investigated and could not be assessed as confounding factors influencing the prevalence of URTIs. Several studies have reported that polyphenols, including catechins, affect immune function and enhance the robustness of cellular immune responses [36,37,38,39,40,41,42]. These effects on immune function were not evaluated in this study and could affect the results. Second, although the subjects were prohibited from discussing the beverages and intervention with each other, it is possible that some of the subjects may have known that they were consuming more or less beverage than other subjects. They would not have known, however, if they were consuming a catechins or placebo beverage. Therefore, there might be a bias due to subject’s beliefs and preconceptions. These limitations should be considered potential confounders in the interpretation and generalizability of the results. Additional long-term, large-scale, double-blinded clinical trials on the general population are needed to clarify the clinical effectiveness of catechins for protecting against UTRIs.

## 5. Conclusions

In conclusion, we conducted a randomized, single-blind, placebo-controlled trial and found that ingesting a beverage containing a certain amount of catechins may be effective toward preventing URTIs and symptom relief. These findings are based on a randomized controlled study of healthcare workers, and thus additional long-term, large-scale clinical trials in the general population are needed to evaluate their generalizability. Further studies will help to clarify the potential therapeutic effects of catechins against viruses.

## Figures and Tables

**Table 1:** Contents of catechin beverage and placebo beverages.

Contents	Catechin Beverage	Placebo Beverage
Catechins (mg)	57	0
Epigallocatechin gallate (mg)	20	0
Epigallocatechin (mg)	18	0
Epicatechin gallate (mg)	6	0
Epicatechin (mg)	5	0
Gallocatechin (mg)	4	0
Gallocatechin gallate (mg)	2	0
Catechin gallate (mg)	1	0
Catechin (mg)	1	0
Caffeine (mg)	10	10
Xanthan gum (mg)	100	100

**Table 2:** Baseline characteristics of the subjects.

Variable	High-Catechin Group (*n* = 84)	Low-Catechin Group (*n* = 85)	Placebo Group (*n* = 86)	*p*-Value ^3^
Sex ^1^				
Male	17 (20.2)	27 (31.8)	15 (17.4)	n/a
Female	67 (79.8)	58 (68.2)	71 (82.6)	0.06
Age (years) ^2^	43.1 (1.37)	43.1 (1.30)	43.1 (1.27)	0.98
BMI (kg/m^2^) ^2^	22.9 (0.45)	23.0 (0.46)	22.6 (0.39)	0.75
Non-smoker ^1^	64 (76.2)	70 (82.4)	70 (81.4)	0.56
Full-time employee ^1^	64 (76.2)	70 (82.4)	67 (77.9)	0.60
Daily preventive behavior ^1^				
Hand washing ^1^	80 (95.2)	84 (98.8)	85 (98.8)	0.29
Hand antisepsis ^1^	54 (64.3)	55 (64.7)	51 (59.3)	0.72
Gargling ^1^	50 (59.5)	47 (55.3)	49 (57.0)	0.86
Flu vaccination ^1^	83 (98.8)	79 (92.9)	82 (95.3)	0.18
Green tea drinking habit ^1,4^	62 (73.8)	60 (70.6)	64 (74.4)	0.83
Daily use of public transportation ^1^	19 (22.6)	16 (18.8)	18 (11.9)	0.83

^1^ absolute and relative frequencies; ^2^ mean (SEM); ^3^ chi-squared test was adopted for nominal and categorical variables: Sex, Non-smoker, Full-time employee, Hand washing, Hand antisepsis, Gargling, Flu vaccination, Green tea drinking habit, and Daily use of public transportation. ANOVA was adopted for the continuous variables: Age and BMI. No statistically significant differences were detected between groups for any variable evaluated; ^4^ >100 mL green tea/day. Abbreviations: BMI, body mass index; SEM, standard error of the mean.

**Table 3:** Mean (95%CI) score of self-assessed subjective symptoms on upper respiratory infections among groups.

Variable	Low-Catechin Group (*n* = 24)		High-Catechin Group (*n* = 11)		Placebo Group (*n* = 23)
	Score	*p*-Value ^+^	Score	*p*-Value ^++^	Score
Nasopharyngeal symptoms ^1^	35.5 (16.2–54.8)	0.04	17.2 (4.6–29.7)	0.02	81.0 (40.6–121.4)
Hypo-pharyngeal symptoms ^1^	33.9 (18.0–49.9)	0.08	33.5 (16.8–50.3)	0.18	62.9 (35.6–90.2)
Systemic symptoms ^1^	40.8 (23.5–58.0)	0.79	41.8 (8.2–75.5)	0.89	49.4 (23.2–75.6)

^+^ Dunnett’s post hoc analysis, low-catechin group vs. placebo group; ^++^ Dunnett’s post hoc analysis, high-catechin group vs. placebo group; Scores of nasopharyngeal symptoms indicate manifestations of sneezing, runny nose, or stuffy nose; hypopharyngeal symptoms indicate manifestations of scratchy throat, dry throat, or sore throat; and systemic symptoms indicate manifestations of fatigue, chills, or joint pain due to cold symptoms or headache, respectively. Abbreviations: CI, confidence interval.

**Table 4:** Incidence of adverse events.

Variable	High-Catechin Group (*n* = 84)	Low-Catechin Group (*n* = 85)	Placebo Group (*n* = 86)
Total number of adverse events	24	14	17
Blood clot	0	0	1
Body pain	6	0	2
Broken hand bone	1	0	0
Cervical lymphadenitis	0	0	1
Dizziness	1	1	0
Eczema	0	1	0
Gastrointestinal complaint	12	6	7
Headache	1	0	1
Hearing impairment	1	0	0
Hemorrhoids	0	1	0
High blood pressure	0	1	0
Insomnia	1	0	0
Joint pain	0	1	0
Nettle rash	0	0	1
Nosebleed	0	0	1
Periodontitis	0	1	1
Polyuria	1	0	0
Sores mouth	0	1	2
Sprain	0	1	0
